# Medicinal plants used in Northern Peru for reproductive problems and female health

**DOI:** 10.1186/1746-4269-6-30

**Published:** 2010-11-01

**Authors:** Rainer W Bussmann, Ashley Glenn

**Affiliations:** 1 William L. Brown Center, Missouri Botanical Garden, P.O. Box 299, St. Louis, MO 63166-0299, USA

## Abstract

Infections of the reproductive tract, complications after childbirth, and reproductive problems continue to be a major health challenge worldwide. An impressive number of plant species is traditionally used to remedy such afflictions, and some have been investigated for their efficacy with positive results. A total of 105 plant species belonging to 91 genera and 62 families were documented and identified as herbal remedies for reproductive problems in Northern Peru. Most species used were Asteraceae (9.52%), followed by Lamiaceae and Fabaceae (8.57% and 6.67%). The most important families are clearly represented very similarly to their overall importance in the local pharmacopoeia. The majority of herbal preparations for reproductive afflictions were prepared from the leaves of plants (22.72%), the whole plant (21.97%), and stems (21.21%), while other plant parts were used less frequently. More than 60% of the cases fresh plant material was used to prepare remedies. Over 70% of the remedies were applied orally, while the remaining ones were applied topically. Many remedies were prepared as mixtures of multiple ingredients.

Little scientific evidence exists to prove the efficacy of the species employed as reproductive disorder remedies in Northern Peru. Only 34% of the plants found or their congeners have been studied at all for their medicinal properties. The information gained on frequently used traditional remedies might give some leads for future targets for further analysis in order to develop new drugs.

## Background

According to 1999 WHO estimates reproductive problems, including, 340 million new cases of curable Sexually Transmitted Diseases (STIs; syphilis, gonorrhoea, chlamydia and trichomoniasis) occur annually throughout the world in adults aged 15-49 years. In developing countries, STIs and their complications rank in the top five disease categories for which adults seek health care. Infection with STIs can lead to acute symptoms, chronic infection and serious delayed consequences such as infertility, ectopic pregnancy, cervical cancer and the untimely death of infants and adults [1].

Traditional Medicine (TM) is used globally and is rapidly growing in economic importance. In developing countries, TM is often the only accessible and affordable treatment available. The WHO reports that TM is the primary health care system for 80% of the population in developing countries. In Latin America, the WHO Regional Office for the Americas (AMRO/PAHO) reports that 71% of the population in Chile and 40% of the population in Colombia have used TM. The WHO indicates that in many Asian countries TM is widely used, even though Western medicine is often readily available, and in Japan, 60-70% of allopathic doctors prescribe TMs for their patients [2].

Complementary Alternative Medicine (CAM) is gaining popularity in many developed countries. Forty-two percent of the population in the US have used CAM at least once [3], and the use of at least one of 16 alternative therapies increased from 34% in 1990 to 42% in 1997 [4]. The number of visits to providers of CAM now exceeds by far the number of visits to all primary care physicians in the US [5,6]. The expenses for the use of TM and CAM are exponentially growing in many parts of the world. The 1997 out-of-pocket CAM expenditure was estimated at US$ 2,7 billion in the USA, and the world market for herbal medicines based on traditional knowledge is now estimated at US$ 60 billion [7].

Northern Peru is believed to be the center of the Central Andean Health Axis [8], and traditional medicinal practices in this region remain an important component of everyday life [9-13]. TM is also gaining acceptance by national governments and health providers. Peru's National Program in Complementary Medicine and the Pan American Health Organization recently compared Complementary Medicine to allopathic medicine in clinics and hospitals operating within the Peruvian Social Security System. The results showed that the cost of using Traditional Medicine was less than the cost of Western therapy. In addition, for each of the criteria evaluated -- clinical efficacy, user satisfaction, and future risk reduction -- Traditional Medicine 's efficacy was higher than that of conventional treatments, including fewer side effects, higher perception of efficacy by both the patients and the clinics, and a 53-63% higher cost efficiency of Traditional Medicine over that of conventional treatments for the selected conditions [14]. According to [6], the sustainable cultivation and harvesting of medicinal species is one of the most important challenges for the next few years.

The present study attempts to give an overview on medicinal plant species employed in Northern Peru in traditional remedies for reproductive problems and female health, and compare this use to the western scientific evidence regarding their efficacy.

## Materials and Methods

### Plant Collections

**Figure 1:**
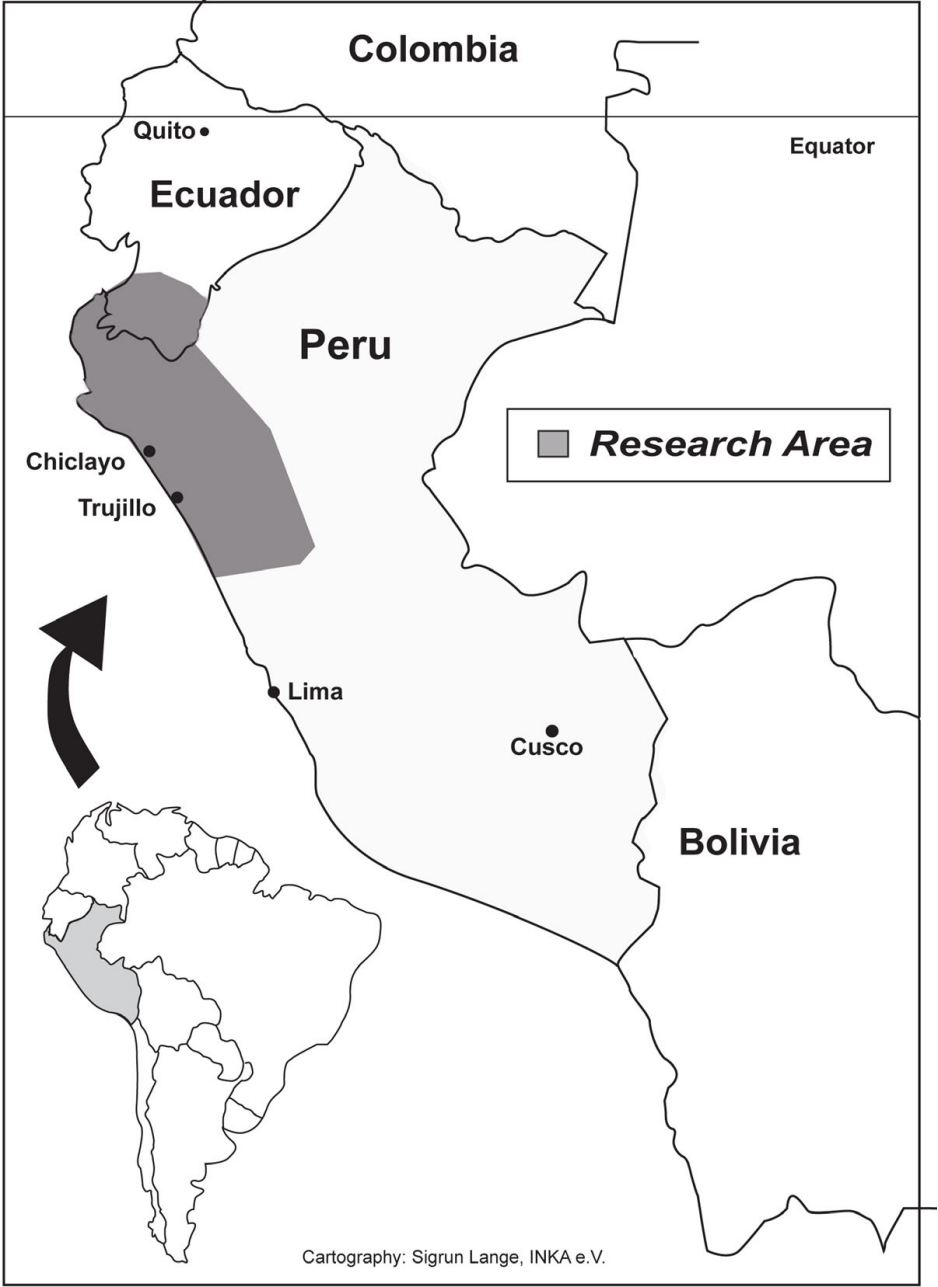
**Location of the study area in Northern Peru**.

Plants for the present study were collected in the field, in markets, and at the homes of traditional healers (*curanderos*) in Northern Peru in 10 2-3 months long field visits between 2001 and 2009, as a larger scale project following initial collections in southern Ecuador (Figure 1). The same 116 informants (healers and market vendors) in the Trujillo and Chiclayo area were repeatedly interviewed during this time, using structured questionnaires. The informants were always provided with fresh (non-dried) plant material, either collected with them, by them, or available at their market stands. The questionnaires did not include any reference as to disease concepts, plant parts or preparations. In contrast, the participants were asked simple questions along the lines "What is this plant used for, which part, which quantity, how is it prepared, are any other plants added to the mixture." All questions were asked in the same order. All informants were of Mestizo origin, and spoke only Spanish as their native language, and all interviews were conducted in Spanish. The study covered the four existing medicinal plant markets of the region, and included all vendors present. All interviews were conducted with the same set of participants. The specimens are registered under the collection series "RBU/PL," "ISA," "GER," "JULS," "EHCHL," "VFCHL," "TRUBH," and "TRUVANERICA," depending on the year of fieldwork and collection location. Surveys were conducted in Spanish by fluent speakers. Surveyors would approach healers, collectors and market vendors and explain the premise for the study, including the goal of conservation of medicinal plants in the area.

Vouchers of all specimens were deposited at the Herbario Truxillensis (HUT, Universidad Nacional de Trujillo), and Herbario Antenor Orrego (HAO, Universidad Privada Antenor Orrego Trujillo). In order to recognize Peru's rights under the Convention on Biological Diversity, most notably with regard to the conservation of genetic resources in the framework of a study treating medicinal plants, the identification of the plant material was conducted entirely in Peru. No plant material collected either in this study in Northern Peru, or the previous study in Southern Ecuador was exported in any form whatsoever.

### Species identification and nomenclature

The nomenclature of plant families, genera, and species follows the *Catalogue of the Flowering Plants and Gymnosperms of Peru *[15] and the *Catalogue of Vascular Plants of Ecuador *[16]. The nomenclature was compared to the TROPICOS database. Species were identified using the available volumes of the *Flora of Peru *[17], as well as [18-20], and the available volumes of the *Flora of Ecuador *[21].

## Results

A total of 105 plant species belonging to 91 genera and 62 families were documented and identified as herbal remedies for reproductive problems in Northern Peru. Most species used were Asteraceae (9.52%), followed by Lamiaceae and Fabaceae (8.57% and 6.67%). Other families were less important, and 44 contributed only one species each to the pharmacopoeia (Table 1). The most important families are clearly represented very similarly to their overall importance in the local pharmacopoeia (Table 1) [9].

**Table 1:** Plants used for reproductive issues in Northern Peru and Comparison of reproductive treatments to the ten most important plant families of the medicinal flora of Northern Peru (after Bussmann & Sharon 2006)

Family	Genera	Species	%	Medicinal flora of Northern Peru (most important families)
				
**Asteraceae**	9	10	9.52	13.64
**Lamiaceae**	7	9	8.57	4.87
**Fabaceae**	6	7	6.67	6.82
**Solanaceae**	2	4	3.81	4.09
**Poaceae**	3	3	2.84	2.33
**Cucurbitaceae**	1	3	2.84	1.75
**Plantaginaceae**	1	3	2.84	
**Amaranthaceae**	2	2	1.92	
**Anacardiaceae**	2	2	1.92	
**Boraginaceae**	2	2	1.92	
**Brassicaceae**	2	2	1.92	
**Euphorbiaceae**	2	2	1.92	2.33
**Olacaceae**	2	2	1.92	
**Rutaceae**	2	2	1.92	
**Dioscoreaceae**	1	2	1.92	
**Geraniaceae**	1	2	1.92	
**Linaceae**	1	2	1.92	
**Passifloraceae**	1	2	1.92	
**Adiantaceae**	1	1	0.95	
**Alstroemeriaceae**	1	1	0.95	
**Amaryllidaceae**	1	1	0.95	
**Apiaceae**	1	1	0.95	2.14
**Apocynaceae**	1	1	0.95	
**Asclepiadaceae**	1	1	0.95	
**Asphodelaceae**	1	1	0.95	
**Balanophoraceae**	1	1	0.95	
**Bignoniaceae**	1	1	0.95	
**Cactaceae**	1	1	0.95	
**Convolvulaceae**	1	1	0.95	
**Cupressaceae**	1	1	0.95	
**Cyperaceae**	1	1	0.95	
**Dipsacaceae**	1	1	0.95	
**Ericaceae**	1	1	0.95	
**Erythroxylaceae**	1	1	0.95	
**Gentianaceae**	1	1	0.95	
**Illiciaceae**	1	1	0.95	
**Isoetaceae**	1	1	0.95	
**Krameriaceae**	1	1	0.95	
**Lauraceae**	1	1	0.95	
**Loganiaceae**	1	1	0.95	
**Loranthaceae**	1	1	0.95	
**Lythraceae**	1	1	0.95	
**Malvaceae**	1	1	0.95	
**Menispermaceae**	1	1	0.95	
**Moraceae**	1	1	0.95	
**Myristicaceae**	1	1	0.95	
**Nyctaginaceae**	1	1	0.95	
**Orchidaceae**	1	1	0.95	
**Oxalidaceae**	1	1	0.95	
**Polygonaceae**	1	1	0.95	
**Polypodiaceae**	1	1	0.95	
**Portulacaceae**	1	1	0.95	
**Proteaceae**	1	1	0.95	
**Ranunculaceae**	1	1	0.95	
**Rosaceae**	1	1	0.95	1.75
**Rubiaceae**	1	1	0.95	
**Thelypteridaceae**	1	1	0.95	
**Thymeleaceae**	1	1	0.95	
**Typhaceae**	1	1	0.95	
**Urticaceae**	1	1	0.95	
**Valerianaceae**	1	1	0.95	
**Verbenaceae**	1	1	0.95	
**Lycopodiaceae**	0	0	0.00	1.95
**TOTAL**	**91**	**105**	**100**	

The majority of herbal preparations for reproductive issues were prepared from the leaves of plants (22.72%), the whole plant (21.97%), and stems (21.21%), while other plant parts were used much less frequently (Table 2). This indicates that the local healers count on a very well developed knowledge about the properties of different plant parts. In almost 62% of the cases fresh plant material was used to prepare remedies, which differs little from the average herbal preparation mode in Northern Peru. Over 70% of the remedies were applied orally, while the remaining ones were applied topically. Many remedies were prepared as mixtures of multiple ingredients by boiling plant material either in water or in sugarcane spirit.

**Table 2:** Plant part used

Plant part	%	Species
**Leaves**	22.72	30
**Whole plant**	21.97	29
**Stems**	21.21	28
**Flowers**	9.85	13
**Root**	8.33	11
**Seeds**	6.82	9
**Bark**	4.55	6
**Fruit**	2.27	3
**Latex**	1.52	2
**Wood**	0.76	1

A complete overview of all plants encountered is given in Table 3.

**Table 3:** Species encountered and used in Northern Peru for reproductive problems

Family/Genus/Species	Indigenous name	Plant part used	Admin.	Use	Coll. #
**ADIANTACEAE**					
*Adiantum concinnum *Wild. ex H.B.K.	Culantrillo del Pozo, Culantrillo	Leaves and Stems, fresh or dried	Oral	Menstrual regulation	VFCHL29, TRUBH17, RBU/PL265, JULS149
**AMARANTHACEAE**					
*Alternanthera porrigens *(Jacquin) Kuntze	Sanguinaria, Moradilla, Lancetilla	Whole plant, fresh or dried	Topical	Cleansing womb after childbirth	EHCHL142, ISA56, RBU/PL301, RBU/PL324, EHCHL93, GER117
*Iresine diffusa *H.B.K. ex Willd.	Paja Blanca, Sangrinaria	Whole plant, fresh	Oral	Inflammation of the ovaries, Menstruation symptoms in adolescents	JULS75, ISA62
**ALSTROEMERIACEAE**					
*Bomarea angustifolia *Benth.	Cachuljillo	Whole plant, dried	Oral	Infertility in women	ISA27
**AMARYLLIDACEAE**					
*Eustephia coccinea *Cav.	Tumapara, Pomanpara, Puma Para, Para Para	Bark, fresh or dried	Oral	Inflammation of uterus	RBU/PL313, GER71, EHCHL68
**ANACARDIACEAE**					
*Mauria heterophylla *H.B.K.	Shimir, Tres Hojas, Trinidad, Chacur, Ahimir, Feregreco	Leaves, fresh	1. Oral 2. Topical	1. Inflammation of uterus, Inflammation of the ovaries, Cysts, Fibroids 2. Vaginal cleansing	ISA24, JULS17, EHCHL83
*Schinus molle *L.	Molle, Moy	Bark and Latex, fresh	Topical	Vaginal infection	EHCHL123, JULS196, GER13
**APIACEAE**					
*Petroselinum crispum *(Miller) A.W. Hill	Perejil	Whole plant, fresh	Oral	Regulation of menstrual cycle	ISA80, EHCHL31, ISA117, RBU/PL278, JULS225
**APOCYNACEAE**					
*Thevetia peruviana *(Pers.) Schum.	Mailchin, Maichil, Camalonga, Cabalonga	Seeds, dried	Oral	Menopause	EHCHL162, TRUVan/Erica19, JULS187, EHCHL174, GER225
**ASCLEPIADACEAE**					
*Sarcostemma clausum *(Jacquin) Schultes	Marrajudio	Leaves, Stems, fresh	Oral	Promoting lactation in women after birth	JULS121, GER43
**ASPHODELACEAE**					
*Aloe vera *(L.) Burm f.	Sabila, Zabila, Aloe, Hojas de Sabila, Aloe Vera	Leaves, fresh	Topcial	Vaginal inflammation, Vaginal ulcers, Vaginal cancer	JULS274, GER22, EHCHL165, VFCHL10
**ASTERACEAE**					
*Ambrosia peruviana *Willd.	Altamisa, Marco, Artamisa, Manzanilla del Muerto, Ajenjo, Llatama Negra Malera, Llatama Roja Malera	Leaves and Stems, fresh	Topical	After birth to reduce inflamation and prevent spasms in the woman's womb	JULS108, TRUBH18, RBU/PL370, TRUBH15, JULS90, GER9, GER110
*Artemisia absinthium *L.	Ajenco	Whole plant, preferably Leaves and Stems, fresh	Oral	Menstrual colics, Menstration, Regulating the menstrual cycle	ISA66, RBU/PL363, GER146
*Chuquiraga spinosa *sp. *huamanpinta *C. Ezcurra	Chuquiragua, Huamanpinta	Leaves, dried	Oral	Prostate, Prostate inflammation, Sexual impotence	EHCHL168, TRUBH9, JULS276, RBU/PL373
*Clibadium *cf. *sylvestre *(Aubl.) Baill.	Flor de Novia	Flowers, Leaves and Stems, fresh or dried	Topical	Before marriage	EHCHL80
*Matricaria frigidum *(HBK) Kunth	Manzanilla	Whole plant, fresh or dried	Topical	Inflammation of the vagina	JULS22, EHCHL1, TRUBH7
*Matricaria recutita *L.	Manzanillon, Agua de la Banda, Manzanilla Blanca, Manzanilla Amarga, Manzanilla	Whole plant, fresh	Topical	1. Vaginal cleansing 2. Menstrual colics	JULS192, RBU/PL306, ISA120, ISA76, GER145
*Monactis flaverioides *H.B.K.	Hierba del Susto (Amarillo), Malva, Mocura, Hierba del Susto, Hierba Susto	Stems and Leaves, fresh	1. Topical 2. Oral	1., 2. Vaginal cleansing	EHCHL19, RBU/PL274, TRUVan/Erica7, ISA104, ISA72
*Paranephelius uniflorus *Poepp. & Endl.	Pacha Rosa, Carapa de Chancho	Whole plant, fresh or dried	Oral	Inflammation of the ovaries, Uterus, Inflammation (internal female parts	EHCHL133, JULS125
*Schkuhria pinnata *(Lam.) Kuntze	Canchalagua, Canchalagua (Chica)	Whole plant, fresh	Oral	Menstrual delay, Allergies, Menstruation	RBU/PL266, JULS42, VFCHL27, GER228
*Taraxacum officinale *Wiggers	Diente de Leon, Amargon	Whole plant, fresh	Topical	Ovaries	RBU/PL252, JULS150, GER62, GER189
**BALANOPHORACEAE**					
*Corynaea crassa *Hook. F.	Huanarpo (hembra & macho)	Tuber/Root, fresh	Oral	Fertility, Sexual potency, Male impotence	JULS171, VFCHL52
**BIGNONIACEAE**					
*Crescentia cujete *L.	Higueron	Latex from Leaf, fresh	Topical	Healing of belly button after birth	JULS164
**BORAGINACEAE**					
*Cordia lutea *Lam.	Overo, Flor de Overo, Overal	Flowers, fresh or dried	Oral	Prostate inflammation.	ISA125, EHCHL77, JULS62, GER10
*Tiquilia paronychoides *(Phil.) Rich.	Flor de Arena, Paja de Lagartija, Mano de Raton	Flowers, fresh or dried	Oral	Inflammation of the ovaries	JULS154, EHCHL107, ISA58, GER20
**BRASSICACEAE**					
*Brassica rapa *L.	Nabo	Root, fresh	Topical	Ovaries	JULS201
*Capsella bursa-pastoris *(L.) Medic.	Bolsita del Pastor, Hierba del Pastor, Bolsa de Pastor	Whole plant, fresh or dried	Oral	Prostate	JULS7, VFCHL42, VFCHL12, RBU/PL257, EHCHL6
**CACTACEAE**					
*Opuntia ficus-indica *(L.) Miller	Tuna	Leaves, fresh	Topical	Hair loss	JULS263, GER3
**CONVOLVULACEAE**					
*Ipomoea batatas *(L.) Lamarck	Camote	Whole plant, fresh	Oral	Promoting lactation in women after giving birth	JULS120
**CUCURBITACEAE**					
*Cucumis dipsaceus *Ehrenb.	Jaboncillo de Campo, Jaboncillo, Patito de Campo	Fruits, fresh	Topical	Hair loss (prevention), Stopping baby from breastfeeding	JULS174, GER35, JULS221
*Cucurbita maxina *Duch.	Zapallo	Flowers and joints of Stems, fresh or dried	Oral	Preventing miscarriage	JULS272
*Cucurbita moschata *Duch.	Zapallo	Flowers and joints of Stems, fresh or dried	Oral	Preventing miscarriage	GER32
**CUPRESSACEAE**					
*Cupressus lusitanica *Miller	Cipre, Cipres	Whole plant, fresh	1. Oral 2. Topical	1. Vaginal hemorrhage 2. Hair loss	RBU/PL288, JULS302
**CYPERACEAE**					
*Oreobolos goeppingeri *Sues	Hierba Chupaflor, Hierba de Suerte, Hierba del Carpintero	Leaves, dried	1. Topical	Aphrodisiac	EHCHL149, TRUVan/Erica17, EHCHL67, GER119
**DIOSCOREACEAE**					
*Dioscorea tambillensis *Kunth	Papa Semitona	Tuber, fresh	Oral	Inflammation of ovaries	JULS283, GER140
*Dioscorea trifida *L.f.	Papa Madre, Papa Pacta	Tuber, fresh	1. Oral 2. Topical	1. Uterus disease and discharge, Cysts, Cancer of the Uterus, Inflammation of the ovaries, Vaginal discharge, 2. Fungus, Vaginal cleansing, Cancer of the Uterus	JULS214, EHCHL40, JULS212, GER142, JULS213
**DIPSACACEAE**					
*Scabiosa atropurpurea *L.	Ambarina, Ambarina Negra, Flor de Ambarina	Flowers, fresh	1. Oral 2. Inhaled	Menstrual regulation	JULS100, EHCHL111, RBU/PL372, ISA50
**ERICACEAE**					
*Bejaria aestuans *L.	Pullunrosa, Cadillo, Payama, Hierba de la Postema, Purenrosa, Rosada, Hierba del buen querer	Flowers, Leaves and Stems, fresh or dried	1. Oral	Prostate, Menstrual regulation, Inflammation of uterus, Cysts, Inflammation of ovaries, Inflammation of the womb, Uterus, Menstrual pain	VFCHL22, JULS50, EHCHL39, ISA114, ISA43, JULS234, GER121
**ERYTHROXYLACEAE**					
*Erythroxylon coca *Lam.	Coca	Leaves, dried	Oral	Induce child birth, Strength for woman during childbirth, Helping delivery of newborn	JULS144, GER201
**EUPHORBIACEAE**					
*Chamaesyce hypericifolia *(L.) Millspaugh	Lecherita, Lechera	Whole plant, fresh	Oral	Promoting lactation in women after birth	JULS67, GER41
*Manhiot esculenta *Crantz	Yuca	Tuber, fresh	Oral	Vaginal infection, Vaginal discharge	GER192
**FABACEAE**					
*Caesalpinia spinosa *(Molina) Kuntze	Tara, Talla, Chanchalagua	Seeds pods, fresh or dried	Topical	Fungus, Inflammation of ovaries, Inflammation of uterus, Inflammation of the vagina	ISA55, EHCHL27, VFCHL21, JULS255, GER143
*Desmodium molliculum *(H.B.K.) DC.	Pie de Perro, Pata-Perro, Pata de Perro, Chancas de Comida, Muña, Manayupa	Whole plant, fresh or dried	Topical	Inflammation of the ovaries, Inflammation of the womb	JULS41, RBU/PL268, GER135, JULS44, EHCHL109, RBU/PL256
*Indigofera suffruticosa *Miller	Añil	Stems, fresh	Oral	Cleaning of the woman, Expelling placenta from woman after giving birth	GER198
*Inga edulis *C. Martius	Huaba, Pacae, Guava, Pacai	Flowers, fresh	Topical	Hair growth	JULS168, JULS304, GER17
*Inga feuillei *DC.	Huaba, Pacae, Guava, Pacai	Flowers, fresh	Topical	Hair growth	JULS168, JULS304, GER17
*Mimosa nothacacia *Barneby	Uña de Gato de la Costa	Bark, dried	Topical	Anus cyst, Vaginal pimples, Anal pimples	JULS265, GER199
*Prosopis pallida *(H. & B. ex Willd.) H.B.K.	Algarrobo	Seeds, dried	Oral	Sexual potency	JULS97, GER8
**GENTIANACEAE**					
*Gentianella bruneotricha *(Gilg.) J.S. Pringle.	Anga Macha	Whole plant, fresh	Oral	Infection of the uterus, After giving birth	JULS282
**GERANIACEAE**					
*Pelargonium odoratisimum *(L.) L'Herit.	Malva de Oro, Malva de Olor, Malva Olorosa	Whole plant, fresh or dried	Oral	Inflammation of the ovaries, Inflammation of the womb	TRUVan/Erica14, TRUBH6, EHCHL89, JULS188
*Pelargonium roseum *Willd.	Geranio	Flowers and Leaves, fresh	Oral	Hemorrhages, Uterus pain, Inflammation of the uterus	JULS84
**ILLICIACEAE**					
*Illicium verum *Hook. f.	Anis Estrella	Seeds, dried	Oral	Expel residues of feces in stomach of newborn babies	JULS102
**ISOETACEAE**					
*Isoetes andina *R. & P.	Piri Piri	Stems, fresh	Oral	Male impotence	ISA100
**KRAMERIACEAE**					
*Krameria lappacea *(Dombey) Berdet & B. Simpson	Ratania, Raima	Leaves and Root, fresh	Oral	Inflammation of the ovaries	JULS53
**LAMIACEAE**					
*Lepechinia meyenii *(Walpers) Epling	Salvia, Salvia Real	Whole plant, fresh or dried	1. Oral 2. Topical	1. Menstruation 2. Hair loss	RBU/PL303, VFCHL17, ISA91
*Mentha spicata *L.	Hierba Buena, Hierba Buena Silvestre, Menta	Whole plant, fresh	Oral	Aphrodisiac	RBU/PL308, EHCHL74, RBU/PL267, JULS72, VFCHL3, JULS20, GER15, GER134, JULS20
*Ocimum basilicum *L.	Albaca Mistura, Albaca Negra, Albaca, Albaca Morada, Albahaca (costa)	Whole plant, fresh	Oral	1. To promote dialation of the uterus, Hasten delivery, Preventing infections related to birth, Refreshing womb, Reducing inflammation after birth 2. After birth	JULS54, EHCHL48, VFCHL13, RBU/PL284, TRUVan/Erica8, GER191
*Origanum majorana *L.	Mejorana	Leaves and Stems, fresh	Oral	Menstration	EHCHL88, JULS19, RBU/PL317, GER165
*Origanum vulgare *L.	Oregano	Leaves and Stems, fresh or dried	Oral	Menstrual cramps, Menstration, Lower stomach cramps related to PMS	JULS205, GER114
*Rosmarinus officinalis *L.	Romero, Romero Castilla	Leaves, fresh or dried	Topical	Hair loss	RBU/PL329, ISA78, TRUBH11, EHCHL3, JULS27, VFCHL2, ISA105
*Salvia discolor *H.B.K.	Palmeras (Chica), Llatama, Yatama	Stems, fresh	1. Topcial 2. Oral	1. Preventing infections related to birth, Fright/Susto in children 2. Preventing infections related to birth	ISA93, ISA151(93a), ISA25
*Salvia officinalis *L.	Salvia	Whole plant, fresh or dried	Oral	Control and regulate menstrual cycle	JULS241
*Satureja pulchella *(H.B.K.) Briquet	Panizara, Panisara	Leaves, fresh or dried	Oral	Menstrual delay	GER148, JULS43
**LAURACEAE**					
*Persea americana *Mill.	Palta	Seeds, fresh	Oral	Contraceptive, Sterilization for women only	JULS211, GER18
**LINACEAE**					
*Linum sativum *L.	Linaza	Seeds, dried	Oral	Inflammation of the prostate	EHCHL1599
*Linum usitatissimum *L.	Linaza	Seeds, dried	Oral	Inflammation of the prostate	JULS185, GER139
**LOGANIACEAE**					
*Buddleja utilis *Kraenzl.	Flor Blanca	Flowers, fresh or dried	Oral	Menstruation, Inflammation of the womb, Ovarian cysts, Inflammation of uterus	RBU/PL333, EHCHL38, ISA60, JULS155, GER136
**LORANTHACEAE**					
*Tristerix longibracteatus *(Des.) Barlow & Wiens	Suelda con Suelda	Whole plant, dried	Oral	Vaginal discharge (white or yellow)	JULS296, GER74
**LYTHRACEAE**					
*Cuphea strigulosa *H.B.K.	Lancetilla, Gacetilla, Sanguinaria, Gansetilla, Hierba del Toro	Leaves and Stems, fresh	Oral	Discharges	GER104, EHCHL35, VFCHL34, JULS33, ISA51, RBU/PL259, EHCHL43, JULS59, ISA53, GER147
**MALVACEAE**					
*Malva sylvestris *L.	Malva (Chica), Malva Blanca	Leaves and Stems, fresh or dried	Topical	Vaginal cleansing	VFCHL49, EHCHL29
**MENISPERMACEAE**					
*Abuta grandiflora *(Mart.) Sand.	Abuta (male and female)	Root and Stems, fresh or dried	Oral	Contraceptive	JULS88, RBU/PL312
**MORACEAE**					
*Brosmium rubescens *Taubert	Palo Sangre, Palo de la Sangre, Ablita	Wood and Bark, fresh or dried	Oral	1. Fertility, Sexual potency 2. Haemorrhages (prevention and healing	JULS209, ISA49, EHCHL64, RBU/PL311, GER86, EHCHL62
**MYRISTICACEAE**					
*Myristica fragrans *L.	Nuez Moscada, Ajonjoli	Seeds, dried	Oral	Fertility, Sexual potency	RBU/PL385, EHCHL155, JULS292, GER197
**NYCTAGINACEAE**					
*Mirabilis jalapa *L.	Buenas Tardes	Root, fresh	Oral	Prostate, Pre-prostate cancer	JULS116, GER185
**OLACACEAE**					
*Heisteria acuminata *(H. & B.) Engler	Chuchuasi, Chuchuhuasi	Bark, fresh or dried	Oral	Sexual potency	RBU/PL287, JULS138, GER164
*Ximenia americana *L.	Limoncillo	Whole plant, fresh or dried	Oral	Menstrual regulation	JULS184
**ORCHIDACEAE**					
*Aa paleacea *(H.B.K.) Rchb. f.	Hierba de la Soledad, Hierba Sola	Leaves, fresh	Oral	Contraceptive, Sterilization of women	ISA141, EHCHL75
**OXALIDACEAE**					
*Oxalis tuberosa *Molina	Oca Rosada	Tuber, fresh	Oral	Sexual potency	JULS203
**PASSIFLORACEAE**					
*Passiflora quardrangularis *L.	Hojas de Tumbo	Leaves, fresh	Oral	Menstrual pain	EHCHL135
*Passiflora *sp.	Chulgan	Leaves and Stems, dried	Oral	Promoting vaginal dilation during childbirth.	JULS279
**PLANTAGINACEAE**					
*Plantago major *L.	Llantén	Leaves, fresh	Topical	Vaginal cleansing	VFCHL50, EHCHL11, TRUVan/Erica13
*Plantago sericea *R. & P. var. *lanuginosa *Grieseb.	Pajilla Blanca	Whole plant, fresh or dried	Oral	Vaginal discharge	JULS207
*Plantago sericea *R. & P. subsp. *sericans *(Pilger) Rahn	Paja Blanca	Stems, fresh or dried	Oral	Ovarian pain, Inflammation of the ovaries, Inflammation of the womb	RBU/PL335, EHCHL96
**POACEAE**					
*Cynodon dactylon *(L.) Persoon	Grama Dulce	Stems, dried	Oral	Cysts of the ovary, Cysts of the uterus, Uterus, Fibroids, Uterus prolapse	ISA61, JULS73, ISA106, GER151
*Saccharum officinarum *L.	Azucar de Caña, Caña de Azucar, Caña Dulce	1. Fresh sugar 2. Stems, fresh	1. Topical 2. Oral	1. Aphrodisiac 2. Inflammation of the prostate	VFCHL4, JULS123, GER208
*Triticum sativum *L.	Trigo	Seeds, dried	Topical	Vaginal infection, Vaginal discharge	GER182
**POLYGONACEAE**					
*Rumex crispus *L.	Acelga, Lengua de Vaca, Hojas de Mala Hierba	Whole plant, fresh	1. Oral 2. Topical	1. Infection of the uterus 2. Inflamation (internal woman parts), Vaginal inflammation	JULS70, EHCHL173
**POLYPODIACEAE**					
*Polypodium crassifolium L*.	Lengua de Ciervo, Calaguala	Stems, fresh	Oral	Prostate	EHCHL71, TRUBH38, RBU/PL331, RBU/PL332, JULS52, JULS303
**PORTULACACEAE**					
*Portulaca villosa *H.B.K.	Verdolaga	Root and Stems, fresh	Topical	Hair loss	GER171
**PROTEACEAE**					
*Oreocallis grandiflora *(Lam.) R.Br.	Rumilanche, Bunbun, Huaminga	Leaves and Stems, fresh or dried	Oral	Inflammation of the ovaries, Inflammation of uterus	EHCHL127, JULS31, ISA28, ISA70
**RANUNCULACEAE**					
*Laccopetalum giganteum *(Wedd.) Ulbrich	Huamanripa, Pacra, Flor de Guarmarya	Leaves, fresh or dried	Oral	Fertilization (Heat Ovaries)	VFCHL53, RBU/PL321, EHCHL42, JULS284, GER162
**ROSACEAE**					
*Sanguisorba minor *Scop.	Pimpinela, Flor de Overa	Whole plant, fresh	Oral	Menstrual regulation	EHCHL117, TRUBH35, RBU/PL262, ISA57, JULS25, ISA147(103a), VFCHL20, GER170
**RUBIACEAE**					
*Cinchona officinalis *L.	Cascarilla, Quinuagiro	Bark, dried	Oral	Fertility, Sexual potency	RBU/PL314, JULS127, ISA19, GER167
**RUTACEAE**					
*Ruta graveolens *L.	Ruda, Ruda (Macho y Hembra), Hierba del Quinde	Whole plant, fresh	1. Oral 2. Topical	1. Abortion 2. Aphrodisiac.	ISA152, JULS1, TRUVan/Erica20, EHCHL128, VFCHL16, ISA145(108a), GER24
*Pouteria lucuma *(R. & P.) Kuntze.	Lucuma	Fruit, fresh	Oral	Promoting lactation on women after giving birth	JULS186
**SOLANACEAE**					
*Cestrum auriculatum *L'Herit	Hierba Santa, Agrasejo	Leaves, fresh or dried	Topical	Preventing spasms after giving birth, Warming women	JULS166, RBU/PL281, EHCHL172, ISA122, GER174, EHCHL102
*Cestrum strigilatum *R. & P.	Santa María	Flowers, leaves and Stems, fresh or dried	Oral	Control and regulate menstrual cycle	JULS245
*Cestrum undulatum *R. & P.	Santa María	Flowers, leaves and Stems, fresh or dried	Oral	Control and regulate menstrual cycle	JULS246
*Solanum tuberosum *L.	Chuno de Papa	Tuber, dried	Oral	After childbirth complications	JULS140, JULS141
**THELYPTERIDACEAE**					
*Thelypteris *cf. *scalaris *(Christ.) Alton	Helecho Macho	Whole plant, fresh or dried	Oral	Contraceptive	JULS291
**THYMELEACEAE**					
*Daphnopsis weberbaueri *Domke	Los Cholitos, Cholitos	Seeds, dried	Oral	Infertility in women	EHCHL153, JULS137, GER216
**TYPHACEAE**					
*Typha angustifolia *L.	Chante	Stems, dried	Oral	Prostate	ISA45
**URTICACEAE**					
*Pilea microphylla *(L.) Lieberman	Contra Hierba	Whole plant, fresh	Oral	Prostate, Cysts	RBU/PL282, EHCHL33
**VALERIANACEAE**					
*Phyllactis rigida *(R. & P.) Persoon	Hornamo Estrella, Siete Sabios, Valeriana Estrella, Valeriana, Hierba de la Estrella	Stems, fresh	Oral	Menopause	EHCHL163, TRUBH30, JULS57, EHCHL44, JULS46, ISA137, RBU/PL365, RBU/PL355, GER187
**VERBENACEAE**					
*Lantana scabiosaefolia *H.B.K.	Mastrando, Mastrante	Leaves and Stems, fresh or dried	Oral	Cold of the ovaries, Menstruation, Women after childbirth to avoid colds	VFCHL51, GER6

## Discussion

Little scientific evidence exists to prove the efficacy of the species employed as reproductive disorder remedies in Northern Peru. Only 34% of the plants found or their congeners have been studied at all for their medicinal properties. *Aloe *spp. are known to have oestrogenic activity [22,23]. [24] reported that *Artemisia *spp. had effects on female health amongst the Cumash. A variety of other Asteraceae has been shown to be used against menopausal symptoms (*Clibadium*: [25]; *Matricaria*: [26-28]; *Taraxacum*: [29,30]. [23] found hormonal effects in *Cordia *sp., while [31-35] reported on anti-fertility effects of *Dioscorea *sp. *Cupressus *sp. are well known abortifacients (e.g. [36]), while pumpkin seed oil showed testosterone-inhibitory effects (e.g. [23,37-39]). *Chamaesyce *sp. showed promise in the treatment of male infertility, while *Mimosa *sp. on the contrary are used to reduce spermal fertility [23,40].

A wide range of Lamiaceae have been shown to exhibit contraceptive efficacy, and the same species are used in Peru for similar purposes (*Mentha *spp.: [41-44]; *Ocimum *spp.: [45-48]; *Origanum majorana*: [44,49,50]; *Rosmarinus officinalis*: [40]). Similar efficacy has been shown for *Sanguisorba officinalis *[51], and *Ruta graveolens *[23,52-55].

Various species of *Passiflora *have aphrodisiac activity [56-60], and *Myristica fragrans *as well as *Syzygium aromaticum *[61,62], and extracts of *Lantana camara *[63,64] and *Pilea *spp. [23] fulfil the same purpose, while *Portulaca oleracea *showed efficacy in relieving uterine bleeding [65,66].

## Conclusions

Infections of the reproductive tract, complications after childbirth, and reproductive problems continue to be a major health challenge worldwide. An impressive number of plant species is traditionally used to remedy such afflictions, and some have been investigated for their efficacy with positive results. An often-limiting factor to these investigations is lack of comprehensive ethnobotanical data to help choose plant candidates for potency/efficacy tests. Since the plant parts utilized in preparation of the remedies are reported in this survey, it serves as an indication of species that may need further ecological assessment on their regeneration status.

The results of this study show that both indigenous and introduced species are used for the treatment of reproductive system problems. The information gained on frequently used traditional remedies might give some leads for future targets for further analysis in order to develop new drugs. However, more detailed scientific studies are desperately needed to evaluate the efficacy and safety of the remedies employed traditionally.

## Declaration of competing interests

The authors declare that they have no competing interests.

## Authors' contributions

RB collected/identified plant material analysis of the data as well as writing the manuscript. AG conducted fieldwork, data analysis and manuscript composition. Both authors have read and approved the final manuscript
